# Obesity in Recovery From Influenza‐Like Illness and Effectiveness of Oseltamivir

**DOI:** 10.1111/irv.70185

**Published:** 2025-11-10

**Authors:** Oanh Ngoc Nguyen, Nathaly Garzón‐Orjuela, Alike W. van der Velden, Christopher C. Butler, Akke Vellinga

**Affiliations:** ^1^ CARA Network, School of Public Health, Physiotherapy and Sports Science University College Dublin Dublin Ireland; ^2^ National Centre for Pharmacoeconomics Dublin Ireland; ^3^ Old Stone Building, Trinity Centre for Health Sciences, St. James's Hospital Dublin 8 Ireland; ^4^ Julius Center for Health Sciences and Primary Care University Medical Center Utrecht, Utrecht University Utrecht the Netherlands; ^5^ Nuffield Department of Primary Care Health Sciences University of Oxford Oxford UK

**Keywords:** influenza‐like illness, obesity, oseltamivir, recovery adults, severity

## Abstract

**Background:**

Results from observational studies have shown that obesity has a mild to moderate association with influenza‐like illness (ILI) severity and hospitalization risk. Using data from the ALIC^4^E Randomized Clinical Trial (RCT), this study investigated the relationship between obesity and ILI severity, time to recovery, and oseltamivir effectiveness in the obese population.

**Methods:**

A total of 2622 adults (≥ 18 years old) from the ALIC^4^E RCT were categorized by body mass index (BMI) into under/normal weight (BMI < 25 kg/m^2^), overweight (BMI ≥ 25 kg/m^2^ and < 30 kg/m^2^), and obesity (BMI ≥ 30 kg/m^2^). ILI symptom severity, time to recovery, and oseltamivir effectiveness were assessed across these weight groups.

**Results:**

At presentation, ILI symptom severity was not different between weight groups. However, time to recovery was longer for obese patients compared to under/normal weight patients, with adjusted HR 0.88 (95% CI 0.79–0.99). The mean time to recovery was 6.6 days (95% CI 6.0–7.1) for obese patients, 6.2 days (95% CI 5.8–6.6) for overweight, and 5.7 days (95% CI 5.4–6.1) for under/normal weight patients. Obese patients had similar benefits from oseltamivir treatment compared to under/normal weight patients, with an average of 0.8 days gained from oseltamivir (95% CI 0.7–1) and 0.5 days (95% CI 0.4–0.7), respectively.

**Conclusions:**

ILI symptom severity at presentation was equally distributed between the three weight groups. However, their time to recovery was approximately 1 day longer compared to under/normal weight patients. The effectiveness of oseltamivir appears to be similar between the two groups.

## 1 Introduction

Seasonal influenza appears in annual epidemics, contributing significantly to the disease burden [1]. Individuals infected with influenza or other respiratory viruses, such as rhinovirus, SARS‐CoV‐2, adenovirus, and respiratory syncytial virus, commonly seek medical attention with symptoms resembling influenza‐like illness (ILI) [2]. ILI is an acute respiratory infection, presenting abruptly with a fever ≥ 38°C and symptoms such as dry cough, headache, sore muscles, or sore throat [3]. Current studies have shown that obesity may influence the susceptibility and complicate the course of illness of several infectious diseases, including respiratory tract infections, those resembling influenza [4, 5].

Cocoros et al. and Guerrisi et al. found in observational studies that ILI patients with obesity had slightly increased odds of severe ILI during influenza seasons, suggesting an association with hospitalization due to ILI [6, 7]. Indeed, the risk increased significantly with morbidly obese patients, as Moser et al. reported an 18.4‐fold increase in odds of hospitalization for morbidly obese patients compared to normal weight adults [8]. Furthermore, in vaccinated adults, Neidich SD et al. found that despite equivalent serological responses to the vaccine, vaccinated obese adults had double the risk of influenza and ILI compared to vaccinated healthy counterparts. This risk remained consistent after adjusting for vaccine year, age, sex, and smoking status [9].

Regarding antiviral treatment, guidelines have recommended the early administration of neuraminidase inhibitors (NAIs) such as oseltamivir and inhaled zanamivir for high‐risk populations, including morbidly obese ILI patients [10]. However, in primary care, where most ILI patients are managed, data suggested a low percentage of NAI prescriptions for ILI outpatients, particularly during seasonal epidemics. Additionally, there is a low proportion of ILI patients, including those with obesity, seeking healthcare promptly [11, 12, 13, 14].

A few randomized clinical trials (RCTs), such as the ALIC^4^E RCT, have evaluated antiviral treatment in ILI patients; however, obese adults have not been specifically assessed in the analyses [15, 16]. Most evidence regarding the severity of ILI in obese patients, as well as the effectiveness of antiviral treatment for this population, is provided by observational studies to date. This study is a post hoc subgroup analysis of the ALIC^4^E RCT, aimed to utilize data from this trial to investigate the relationship between obesity and ILI, compare the time to recovery between obese and nonobese patients with ILI, and assess the effectiveness of oseltamivir in treating ILI in the obese population [17].

## 2 Methods

ALIC^4^E (trial registration number ISRCTN27908921) was an open‐label, pragmatic, adaptive RCT conducted between January 15, 2016, and April 12, 2018, over three consecutive influenza seasons. Patients were enrolled in the trial when they presented at their general practitioner (GP) with ILI symptoms or sought an appointment or advice about their symptoms via telephone, at medical practices that were part of 209 primary care practices of 21 research networks in 15 European countries [15].

### 2.1 Eligible Criteria

Patients eligible for the trial were aged at least 1 year, presented with ILI symptoms, were willing and able to give informed consent, and agreed not to take antiviral agents apart from the study's antiviral agent. Patients were excluded if they had chronic kidney failure (e.g., recorded in GP record or an estimated glomerular filtration rate of less than 60 mg/L), had severe liver impairment, were immunocompromized, required immediate antiviral therapy, or urgent hospital admission. Patients with known allergies to oseltamivir or any trial medication, scheduled for elective surgery or a procedure requiring general anesthesia for the next 2 weeks, or requiring live vaccine for the next 7 days were also excluded [15].

A total of 3266 patients were recruited in the trial and randomized in a 1:1 ratio, using blocks of two, four, or six to receive either usual care according to GPs' preferences or oseltamivir plus usual care. Randomization was stratified by age, GP‐related overall ILI severity, comorbidities, and duration of symptoms at study enrolment. The methods and results of this study were published elsewhere [15].

### 2.2 Data Analysis

In this post hoc subgroup analysis, only adults who were at least 18 years of age at the time of study enrolment were included. Based on the World Health Organization Obesity Classification, body mass index (BMI) was calculated using weight (kg)/ [height (m^2^)] and categorized into three groups: obesity (BMI ≥ 30 kg/m^2^), overweight (25 kg/m^2^ ≤ BMI < 30 kg/m^2^), and under/normal weight group (BMI < 25 kg/m^2^). Within the last category, patients with a BMI < 18.5 kg/m^2^ were classified as underweight [18].

ILI symptom severity was rated by patients at the study enrolment. This included 15 self‐rated variables covering respiratory symptoms (running/congested nose, sore throat, headache, cough, and shortness of breath), gastrointestinal symptoms (diarrhea, nausea/vomiting, and abdominal pain), general malaise symptoms (low energy, not sleeping well, dizziness, and general unwell), and inflammatory symptoms (fever, muscle ache, and sweats/chills) [15]. To describe patients' individual ILI symptom severity at presentation, an ILI severity score was created using these 15 self‐rated variables. Symptom severity at presentation was rated by patients on a scale from 0 to 3, where 0 signified no problem and 3 indicated a major problem. The possible score could range from 0 to 45. Differences between the three weight groups were assessed by the one‐way ANOVA (analysis of variance) test. Each symptom was later examined separately by the chi‐square test.

ILI symptom severity was also rated by general practitioners (GPs) at the study enrolment as the GP‐related overall ILI severity. This was categorized into mild, moderate, and severe. To assess the GP‐related overall ILI severity, a chi‐square test for trend was used.

The primary outcome of the study was time to recovery, defined as the return to usual activities with fever, headache, and muscle aches rated as minor or no problem, using data on these symptoms from the patients' daily diary [15]. The time to recovery and effectiveness of oseltamivir were assessed between under/normal weight, overweight, and obesity groups. A Cox proportional hazard model was used, adjusting for sex, age group, duration of symptoms at enrolment, GP‐related overall ILI severity, and comorbidities. A formal test for effect modification by BMI category was conducted using interaction terms in the Cox model. The effect of oseltamivir was further assessed within each subgroup (under/normal weight, overweight, and obesity groups). Sensitivity analyses were conducted, excluding underweight patients in the under/normal weight group, to assess the robustness of the primary findings. A *p* value less than 0.05 was considered statistically significant. Data analysis was performed using R version 4.3.1 [19].

## 3 Ethical Approval

This study was approved by the University College Dublin Undergraduate and Taught Masters Research Ethics Committee (UTMREC‐23‐10‐NGUYEN‐VELLINGA) on January 11, 2024. The ALIC^4^E RCT was approved by the ethics committees and regulatory authorities in all 15 participating countries [15].

## 4 Results

### 4.1 Characteristics of the Study Population

A total of 2622 adults were included in the analysis, with BMI data missing for 19 adults (0.7%). The remaining 2603 adults were categorized into the under/normal weight group (1284 adults), the overweight group (837 adults), and the obesity group (482 adults). Table 1 presents the characteristics of adult patients in this study. The majority of patients were aged between 18 and 65 years. Notably, there were significantly more females in the under/normal weight and obesity groups compared to the overweight group. Furthermore, adult patients in the overweight and obesity groups had more comorbidities than those in other groups. Particularly, there were more overweight and obese adults having heart disease, diabetes, chronic respiratory conditions, stroke, or transient ischemic attack than overweight and under/normal weight adults. When assessing the etiology of ILI in the population, about 70% of adults had a virus identified as the cause of ILI. Of these, approximately 50% were influenza virus and 10% were coronavirus.

**TABLE 1:** Baseline demographic and clinical characteristics of the study population.

	Under/normal weight (*n* = 1284)	Overweight (*n* = 837)	Obesity (*n* = 482)	*p*
**Sex, *n* (%)**				
Male	475 (37.0)	434 (51.9)	190 (39.4)	<0.0001^a^
Female	809 (63.0)	402 (48.1)	292 (60.6)	
**Age, *n* (%)**				
18–65 years	1238 (96.4)	739 (88.3)	419 (86.9)	<0.0001^a^
>65 years	46 (3.58)	98 (11.7)	63 (13.1)	
**Comorbidities, *n* (%)**				
Comorbidities presented	125 (9.7)	155 (18.5)	136 (28.2)	<0.0001^a^
Heart disease	26 (2.0)	58 (6.9)	59 (12.2)	<0.0001^a^
Diabetes	12 (0.9)	27 (3.2)	40 (8.3)	<0.0001^a^
Chronic respiratory condition	58 (4.5)	52 (6.2)	46 (9.5)	0.0003^a^
Hepatic, hematological, neurological, or neurological development condition	11 (0.9)	13 (1.6)	5 (1.0)	0.322^a^
Overnight hospital stay in the preceding year	3 (0.2)	9 (1.1)	1 (0.2)	0.016^a^
Stroke or transient ischemic attack	23 (1.8)	32 (3.8)	16 (3.3)	0.013^a^
**Overall ILI severity rated by GPs**				
Mild	232 (18.1)	171 (20.4)	93 (19.3)	0.650^a^
Moderate	801 (62.4)	497 (59.4)	297 (61.6)	
Severe	252 (19.5)	169 (20.2)	92 (19.1)	
**ILI severity rated by patients, mean (SD)**	20.5 (6.11)	20.4 (6.0)	20.6 (6.13)	0.855^b^
**ILI symptom duration at study enrolment, *n* (%)**				
≤24 h	366 (28.5)	234 (28.0)	123 (25.5)	0.676^a^
>24–48 h	498 (38.8)	328 (39.2)	186 (38.6)	
>48‐72 h	420 (32.7)	275 (32.9)	173 (35.9)	
**Virus presented in samples, *n* (%)**				
No virus	372 (29.3)	251 (30.2)	136 (28.6)	0.827^a^
Virus presented	899 (70.7)	581 (69.8)	339 (71.4)	
**Coronavirus presented in samples, *n* (%)**				
No coronavirus	1147 (90.2)	749 (90)	423 (89.1)	0.759^a^
Coronavirus presented	124 (9.8)	83 (10)	52 (10.9)	
**Influenza virus presented in samples, *n* (%)**				
No influenza virus	612 (48.2)	406 (48.8)	240 (50.5)	0.677^a^
Influenza virus presented	659 (51.8)	426 (51.2)	235 (49.5)	

^a^ Chi‐squared test.

^b^ One‐way ANOVA test.

### 4.2 ILI Symptom Presentation

In each weight group, approximately 60% of patients were rated by their GP as having moderate ILI, while 20% were categorized as having mild ILI and 20% as having severe ILI. From the GPs' overall illness severity rating and patients' individual symptoms rating, there were no significant differences in ILI severities between G patients (Table 1). There were also no differences in the severities per the 13 individual symptoms severity ratings between weight groups, except for cough and shortness of breath, where obese patients had more moderate and severe symptoms than others. Specifically, 32% of overweight patients and 37% of obese patients had severe cough symptoms, while 31% of those in the under/normal weight group reported severe cough. Additionally, 9% of overweight patients and 11% of obese patients had severe shortness of breath compared to 7% in the under/normal weight group.

### 4.3 Time to Recovery From ILI

**FIGURE 1:**
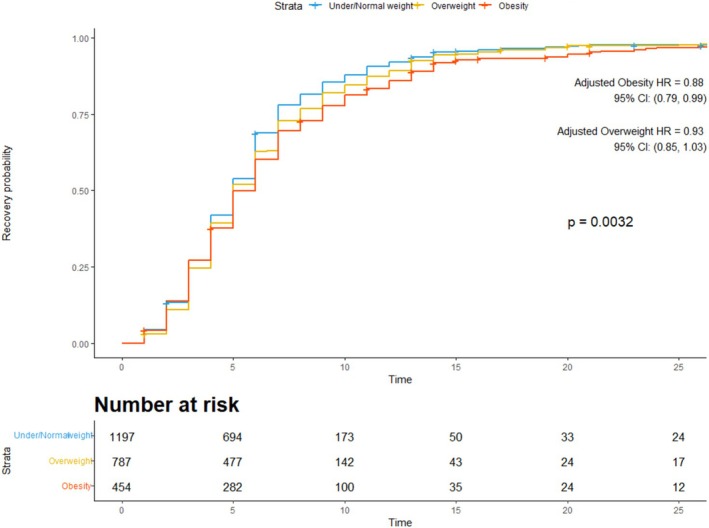
Kaplan–Meier estimates the probability of recovery across time by the three weight groups.

**FIGURE 2:**
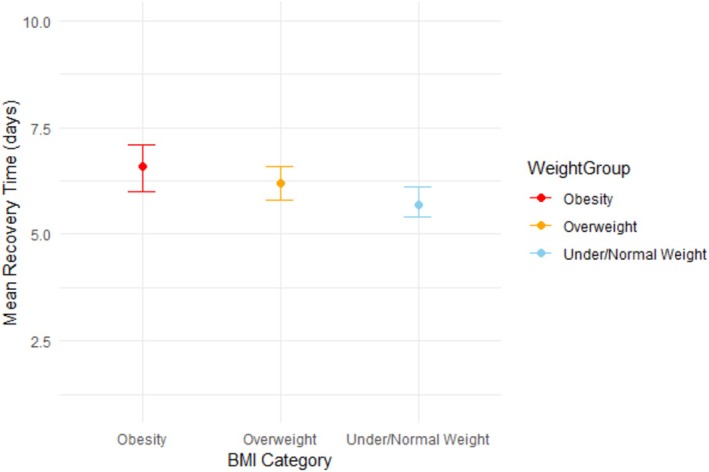
The estimated days of recovery between weight groups from fitting Cox proportional hazard regression model, adjusted for sex, age groups, duration of symptoms at enrolment, comorbidities, and GP‐related overall severity of ILI.

The Cox regression analysis showed a statistically significant difference in time to recovery between the three weight groups. The adjusted HR of the obesity group was 0.88 (95% CI 0.79–0.99) and for the overweight group was 0.93 (95% CI 0.85–1.03), compared to under/normal weight adults as the reference group (Figure 1). The corresponding mean days to recovery were 6.6 days (95% CI 6.0–7.1) for obese adults, 6.2 days (95% CI 5.8–6.6) for overweight adults, and 5.7 days (95% CI 5.4–6.1) for under/normal weight adults (Figure 2).

**FIGURE 3:**
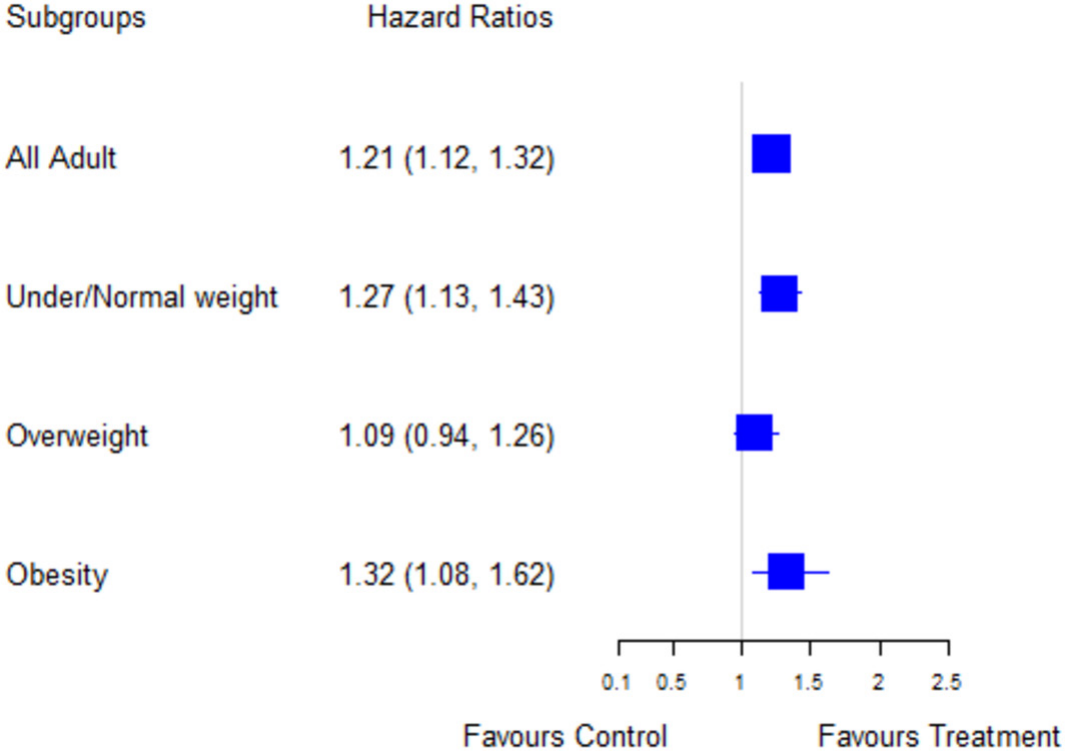
The hazard ratios and 95% CI from fitting Cox proportional hazards regression models of oseltamivir plus usual care versus usual care only between subgroups, stratified by weight groups, adjusted for sex, age group, duration of symptoms at enrolment, comorbidities, and GP‐related overall illness severity.

**FIGURE 4:**
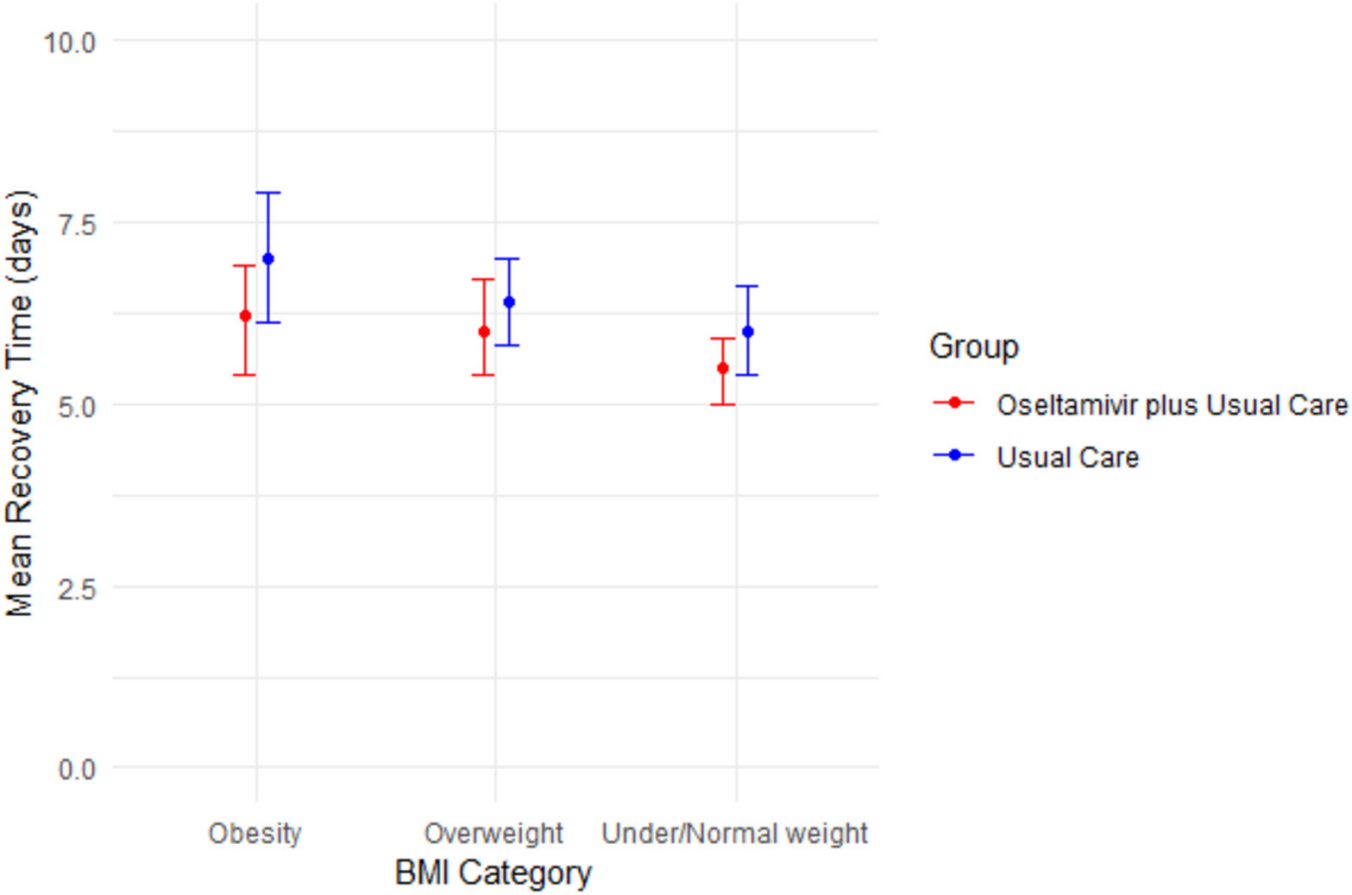
The estimated days of recovery from fitting Cox proportional hazards regression model of oseltamivir plus usual care versus usual care only between weight groups, stratified by weight groups, adjusted for sex, age group, duration of symptoms at enrolment, comorbidities, and GP‐related overall illness severity.

A subgroup analysis stratifying the Cox model according to the BMI categories was performed (Figure 3). For obese patients, the results of stratified subgroup analyses showed an HR of 1.32 (95% CI 1.08–1.62), indicating a shorter recovery time for obese patients who received oseltamivir plus usual care compared with patients receiving usual care only (Figure 3). The time to recovery of those receiving oseltamivir plus usual care was 6.2 days (95% CI 5.4–6.9), and those who received usual care only had the time to recovery of 7 days (95% CI 6.1–7.9) (Figure 4). The estimated mean benefit of oseltamivir for obese patients was 0.8 days (95% CI 0.7–1).

For under/normal weight patients, the stratified subgroup analyses showed an HR of 1.27 (95% CI 1.13–1.43), indicating similar effectiveness of oseltamivir plus usual care as in obese patients (Figure 3). The time to recovery was 5.5 days (95% CI 5–5.9) for those who received oseltamivir plus usual care, compared to 6 days (95% CI 5.4–6.6) for those receiving usual care only (Figure 4). The estimated mean benefit of oseltamivir for under/normal weight patients was 0.5 days (95% CI 0.4–0.7).

As for the overweight group, the difference in time to recovery was nonsignificant with an adjusted HR of 1.09 (95% CI 0.94–1.26) (Figure 3). Time to recovery was 6 days (95% CI 5.4–6.7) for those who received oseltamivir plus usual care, compared to 6.4 days (95% CI 5.8–7) for those receiving usual care only (Figure 4).

Additionally, the sensitivity analyses, in which 76 underweight patients (BMI < 18.5 mg/m^2^) were excluded from the under/normal weight group, showed consistent results with the primary analysis. The time to recovery and effectiveness of oseltamivir between normal weight, overweight, and obesity groups were similar to the findings in the under/normal weight, overweight, and obesity groups.

## 5 Discussion

In this study, obese adults took approximately 1 day longer to recover from ILI, with an average of 6.6 days compared to 5.7 days in under/normal weight adults. The slower recovery in obese adults may be explained by the delayed or blunted antiviral response due to obesity [20]. Obesity delays the clearance of virus load and prolongs the shedding duration, causing long‐term transmission [21]. Some studies suggested that this effect is probably related to the impaired memory T‐cell response in obesity [22, 23].

Obese adults did not present with more severe ILI symptoms than nonobese adults in this study. However, a case‐cohort study found that obese adults aged 20–39 years and those aged 40–59 years had an increased risk of having mild ILI, with adjusted ORs of 1.35 (95% CI 1.16–1.58) and 1.26 (95% CI 1.16–1.43), respectively. Additionally, obese adults aged 40–59 years also showed a higher risk of having severe ILI, with an OR of 2.61 (95% CI 1.31–5.19). The risks were not significant for obese adults aged 20–39 or those above 60 years [6]. A crowdsourced cohort study found that being overweight or obese is a risk factor for developing ILI [7]. Another observational cohort study in hospitalized patients with ILI reported that obese and morbidly obese adults with confirmed influenza had a significantly higher risk of hospitalization [8]. In comparison to this study, these studies were observational. The case‐cohort study that reported an association between obese adults and ILI severity used a different severity definition. ILI severity was determined using codes from the International Classification of Disease, 9th Revision, Clinical Modification (ICD‐9) codes, and was not rated by GP and/or patient themselves. Mild ILI cases were identified by diagnostic codes from outpatient services, including emergency visits, while severe ILI cases were indicated by diagnostic codes from inpatient visits.

This study also found that the effectiveness of oseltamivir in ILI treatment is similar between obese and under/normal weight adults, with an average of 0.8 days gained in the obese group compared to 0.5 days gained in the under/normal weight group. The results from sensitivity analyses confirmed the robustness of this finding. This average day gained is, however, slightly smaller than findings from the original study, where patients receiving oseltamivir plus usual care gained 1.02 days compared to those who received usual care only [15]. This discrepancy may be attributed to the original trial including patients aged 1 year and above, while this study included adult patients only. While specific studies on the effectiveness of oseltamivir in obese humans are lacking, research in influenza‐infected obese mice suggested that a blunted immune response may impair oseltamivir's effectiveness in obese individuals. The benefits of oseltamivir in treating influenza and ILI have been extensively examined in the literature with guidelines strongly recommending prescribing oseltamivir for patients suspected or confirmed to have influenza, who have severe, complicated, or progressive illness, who are hospitalized, or who are at a higher risk for influenza complications, including those who are morbidly obese [10, 24]. This study found that when initiating oseltamivir in the obese population within the treatment window resulted in nearly a day gained in recovery time. Thus, treatment may be recommended for this population if the expected recovery time gained is considered important for patients.

This study used a high‐quality RCT dataset with patients randomized within 72 h since the onset of ILI symptoms; it produced a good starting point for assessing the ILI recovery and the benefits of treatment. Furthermore, ILI symptom severity was assessed from the perspectives of both GPs and patients, which gives a holistic view of ILI symptom burden and recovery.

This study was limited by the paucity of the obese population in the original trial. There were 482 obese patients compared to 1842 normal‐weight patients in our analysis. Initially, the study aimed to analyze the differences between obese and morbidly obese adults; however, only 38 morbidly obese adults were available in the analysis. Thus, these patients were included in the overall obesity group to ensure an adequate sample size. Additionally, as a post hoc subgroup analysis of the ALIC^4^E RCT, which was not randomized by weight, the findings relating to the difference in time to recovery and the effect of oseltamivir by weight group should be interpreted with caution. Future clinical trials could further investigate ILI in the obese population by recruiting and randomizing patients based on weight, thus enhancing the robustness of scientific findings specific to this demographic.

## 6 Conclusions

This study found that ILI symptom severity at presentation was equally distributed between under/normal weight, overweight, and obese patients. However, it takes approximately 1 day longer for obese patients to recover from ILI compared to under/normal weight adults. This delayed recovery underscores the need for timely treatment and additional support necessary for managing obese patients with ILI.

## Author Contributions

**Oanh Ngoc Nguyen:** conceptualization, methodology, writing – original draft, formal analysis, writing – review and editing, visualization. **Nathaly Garzón‐Orjuela:** conceptualization, methodology, formal analysis, writing – review and editing. **Alike W. van der Velden:** investigation, writing – review and editing, data curation, conceptualization. **Christopher C. Butler:** investigation, data curation, conceptualization, writing – review and editing. **Akke Vellinga:** conceptualization, methodology, writing – review and editing, supervision.

## Conflicts of Interest

The authors declare no conflicts of interest.

## Data Availability

The data that support the findings of this study are available from the University Medical Center Utrecht, the Netherlands. Restrictions apply to the availability of these data, which were used under license for this study. Data are available from the corresponding author with the permission of the University Medical Center Utrecht, the Netherlands.
